# Causal Relevance of Lp(a) for Coronary Heart Disease and Stroke Types in East Asian and European Ancestry Populations: A Mendelian Randomization Study

**DOI:** 10.1161/CIRCULATIONAHA.124.072086

**Published:** 2025-04-29

**Authors:** Robert Clarke, Neil Wright, Kuang Lin, Canqing Yu, Robin G. Walters, Jun Lv, Michael Hill, Christiana Kartsonaki, Iona Y. Millwood, Derrick A. Bennett, Daniel Avery, Ling Yang, Yiping Chen, Huaidong Du, Paul Sherliker, Xiaoming Yang, Dianjianyi Sun, Liming Li, Chan Qu, Santica Marcovina, Rory Collins, Zhengming Chen, Sarah Parish

**Affiliations:** Clinical Trial Service Unit, Nuffield Department of Population Health, University of Oxford, UK (R. Clarke, N.W., K.L., R.G.W., M.H., C.K., I.Y.M., D.A.B., D.A., L.Y., Y.C., H.D., P.S., X.Y., R. Collins, Z.C. S.P.).; Department of Epidemiology and Biostatistics, School of Public Health, Peking University Health Science Center, Beijing, China (C.Y., J.L., D.S., L.L.).; Peking University Center for Public Health and Epidemic Preparedness and Response, Beijing, China (C.Y., J.L., D.S., L.L.).; Key Laboratory of Major Diseases (Peking University), Ministry of Education, Beijing, China (C.Y., J.L., D.S., L.L.).; NCDs Prevention and Control Department, Liuyang CDC, China (C.Q.).; Medpace Reference Laboratories, Cincinnati, OH (S.M.).

**Keywords:** coronary disease, East Asian people, European people, heart diseases, lipoprotein(a), stroke

## Abstract

**BACKGROUND:**

Elevated plasma levels of Lp(a) [lipoprotein(a)] are a causal risk factor for coronary heart disease and stroke in European individuals, but the causal relevance of Lp(a) for different stroke types and in East Asian individuals with different Lp(a) genetic architecture is uncertain.

**METHODS:**

We measured plasma levels of Lp(a) in a nested case–control study of 18 174 adults (mean [SD] age, 57 [10] years; 49% female) in the China Kadoorie Biobank (CKB) and performed a genome-wide association analysis to identify genetic variants affecting Lp(a) levels, with replication in ancestry-specific subsets in UK Biobank. We further performed 2-sample Mendelian randomization analyses, associating ancestry-specific Lp(a)-associated instrumental variants derived from CKB or from published data in European individuals with risk of myocardial infarction (n=17 091), ischemic stroke (IS [n=29 233]) and its subtypes, or intracerebral hemorrhage (n=5845) in East Asian and European individuals using available data from CKB and genome-wide association analysis consortia.

**RESULTS:**

In CKB observational analyses, plasma levels of Lp(a) were log-linearly and positively associated with higher risks of myocardial infarction and IS, but not with intracerebral hemorrhage. In genome-wide association analysis, we identified 29 single nucleotide polymorphisms independently associated with Lp(a) that together explained 33% of variance in Lp(a) in Chinese individuals. In UK Biobank, the lead Chinese variants identified in CKB were replicated in 1260 Chinese individuals, but explained only 10% of variance in Lp(a) in European individuals. In Mendelian randomization analyses, however, there were highly concordant effects of Lp(a) across both ancestries for all cardiovascular disease outcomes examined. In combined analyses of both ancestries, the proportional reductions in risk per 100 nmol/L lower genetically predicted Lp(a) levels for myocardial infarction were 3-fold greater than for total IS (rate ratio, 0.78 [95% CI, 0.76–0.81] versus 0.94 [0.92–0.96]), but were similar to those for large-artery IS (0.80 [0.73–0.87]; n=8134). There were weaker associations with cardioembolic IS (0.92 [95% CI, 0.86–0.98]; n=11 730), and no association with small-vessel IS (0.99 [0.91–1.07]; n=12 343) or with intracerebral hemorrhage (1.08 [0.96–1.21]; n=5845).

**CONCLUSIONS:**

The effects of Lp(a) on risk of myocardial infarction and large-artery IS were comparable in East Asian and European individuals, suggesting that people with either ancestry could expect comparable proportional benefits for equivalent reductions in Lp(a), but there was little effect on other stroke types.

**Clinical Perspective**

**What Is New?**

- This study identified 29 variants that were independently associated with lipoprotein(a) [Lp(a)] and explained 33% of variance in Lp(a) in Chinese individuals, with only a modest overlap with Lp(a)-related variants in European individuals.
- East Asian individuals with genetically predicted elevated Lp(a) levels had comparable relative risks of myocardial infarction as European individuals for equivalent differences in Lp(a) levels, despite a lower proportion of East Asian individuals than European individuals having elevated Lp(a) levels.
- The effects of genetically predicted Lp(a) levels on myocardial infarction were 3-fold greater than on ischemic stroke overall, and associations with ischemic stroke were chiefly limited to large-artery subtypes in both populations.

**What Are the Clinical Implications?**

- East Asian and European individuals with elevated plasma levels of Lp(a) can expect comparable proportional benefits from novel Lp(a)-lowering treatments for coronary heart disease prevention.
- The predicted effects of novel Lp(a)-lowering treatments for prevention of stroke are likely to be modest and largely limited to large-artery ischemic stroke subtypes.

Observational and genetic studies conducted in European ancestry populations have demonstrated that higher plasma levels of Lp(a) [lipoprotein(a)] are associated with higher risks of coronary heart disease (CHD),^1–4^ ischemic stroke (IS),^5,6^ and aortic stenosis.^7^ Three large-scale randomized trials involving ≈28 000 participants are assessing the effects on CHD or stroke (or a composite of both outcomes) of novel antisense agents (ASOs) or small interfering RNA (siRNA) therapies that lower Lp(a) levels by >90% (ie, ≈100 nmol/L) by blocking the synthesis of Lp(a).^8–14^ Evaluation of these potent Lp(a)-lowering therapies in both European and East Asian clinical trial populations has prompted interest in comparative studies of the associations of Lp(a) with CHD, stroke and IS subtypes in East Asian and European ancestry populations to guide the interpretation of ongoing trials and inform the design of future Lp(a)-lowering trials for prevention of CHD and stroke in diverse ancestry populations.

Lp(a) is a low-density lipoprotein (LDL)–like particle involving an APOB (apolipoprotein B) covalently linked with an apo(a) [apolipoprotein(a)] particle.^15^ Plasma levels of Lp(a) differ by ≈1000-fold between individuals,^2–4^ due chiefly to genetic variation in the *LPA* locus that encodes the apo(a) particle.^6,15,16^ Apo(a) isoform size varies due to a variable number (range, 2 to 40) of genetically determined kringle IV type 2 (KIV2) repeats between individuals.^15,16^ Utermann^16^ discovered a size polymorphism and an inverse association of plasma Lp(a) levels with apo(a) isoform size that also varied by ancestry.

In European ancestry populations, a meta-analysis of prospective studies demonstrated a monotonic dose–response association of higher levels of Lp(a) with higher risk of CHD,^2^ with concordant findings in Mendelian randomization (MR) studies of genetically predicted Lp(a) and CHD supporting their causal relevance.^4,17,18^ In PROCARDIS (Precocious Coronary Artery Disease), a case–control study in which Lp(a) levels were examined in 3000 European participants with CHD and 3000 European controls, Clarke et al^4^ discovered 2 *LPA* variants (rs10455872 and rs3798220) that were each associated with higher levels of Lp(a) and higher risks of CHD, providing strong support for the causal relevance of Lp(a) for CHD. A genetic score for the combined effects of these 2 *LPA* variants explained 36% of the variance in Lp(a) levels. In a subsequent meta-analysis of prospective studies involving 25 000 adults with genome-wide association study (GWAS) data and Lp(a) levels, Burgess et al^19^ identified 43 independent variants associated with Lp(a) that explained ≈63% of variance in Lp(a), and higher genetically predicted Lp(a) levels were log-linearly and positively associated with higher risks of CHD. Hoekstra et al^20^ also identified 4 variants independently associated with Lp(a) outside the *LPA* locus.

Less is known about the genetic pathogenesis of Lp(a) in East Asian populations, but previous studies, involving a few hundred participants, reported that the rs10455872 and rs3798220 *LPA* variants did not predict Lp(a) levels in East Asian individuals.^21,22^ In a GWAS study of Lp(a) levels in 1403 Chinese adults with CHD, Liu et al^22^ identified 4 GWAS-significant single nucleotide polymorphisms (SNPs) that were independently associated with Lp(a) and together explained 16% of variance in Lp(a) levels. Further GWAS of Lp(a) levels and apo(a) isoform size are required to clarify the genetic determinants of Lp(a) in Chinese individuals. Moreover, comparative MR studies for CHD and stroke (including IS subtypes) are also needed to compare the effect estimates for equivalent differences in Lp(a) levels in East Asian and European populations.

The aims of the current study were to compare the shape and strength of the associations of plasma levels of Lp(a) with myocardial infarction (MI) and stroke types in Chinese adults in a nested case–control study in the China Kadoorie Biobank (CKB)^23^; identify the genetic determinants of Lp(a) levels to discover instrumental variables for MR analyses of MI and stroke types in East Asian individuals; and conduct MR analyses of Lp(a) using ancestry-specific Lp(a)-associated variants with cardiovascular disease (CVD) outcomes (MI, stroke types, and IS subtypes) using combined analyses of CKB with publicly available summary data from GWAS consortia in East Asian and other European populations.^24–27^

## METHODS

### Data Availability

Nongenetic data CKB and analytical methods used in the analysis will be made available to other researchers for replication, but sharing of genetic data is limited by Chinese Regulations on Human Genetic Resources to collaboration with CKB researchers.

Data from baseline, first and second resurveys, and disease follow-up are available under the CKB Open Access Data Policy to bona fide researchers. Sharing of genotyping data is constrained by the Administrative Regulations on Human Genetic Resources of the People’s Republic of China. Access to these and certain other data is available through collaboration with CKB researchers. Details of the CKB Data Sharing Policy are available at www.ckbiobank.org. Genotyping data were exported from China to the Oxford CKB International Coordinating Centre under data export approvals 2014-13 and 2015-39 from the Office of Chinese Human Genetic Resource Administration.

### CKB Study Population

Details of the design and methods used in the CKB study have been reported previously.^23,28^ In brief, the CKB study involved a 12-year follow-up of a prospective study of 512 724 adults 30 to 79 years of age, recruited from 10 regions (5 urban and 5 rural) in China between 2004 and 2008. Baseline characteristics of the participants are shown in the Table. The baseline survey included a laptop-based questionnaire that collected data on sociodemographic and lifestyle factors, medical history and use of medication, and physical measurements. Study participants provided a 10-mL nonfasting blood sample for long-term storage in liquid nitrogen. Follow-up for incident diseases and cause-specific mortality were obtained by electronic linkage to regional death and disease registries and to health insurance records for any episodes of hospitalizations. The CKB study was approved by the relevant local, national, and international ethics committees, and all participants provided written informed consent.^23^

**Table.**
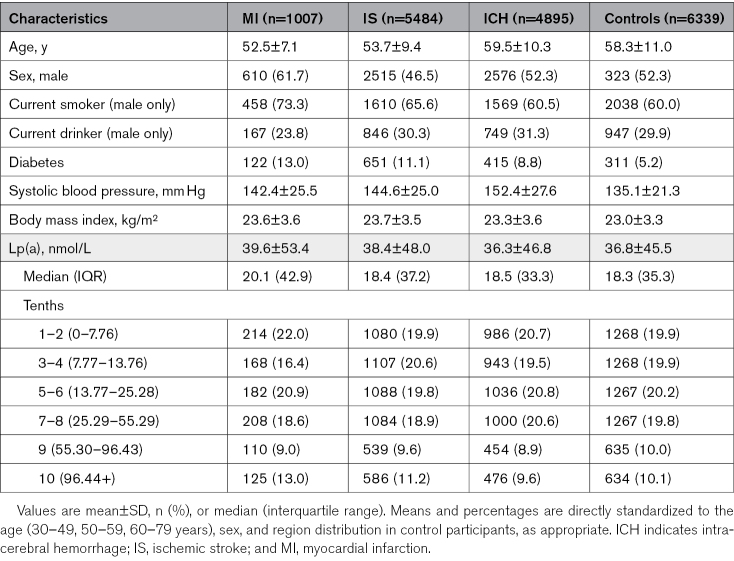
Selected Baseline Characteristics Among Vascular Disease Types versus Controls

### Lp(a) Assays

Plasma levels of Lp(a) in 18 174 participants in a nested case–control study of CHD and stroke types in CKB^23^ were measured in molar units using a polyclonal assay (Denka Seiken) in the Wolfson Laboratory, Nuffield Department of Population Health, University of Oxford.^29^ This polyclonal Lp(a) assay included 5 calibrators and was certified by the Lp(a) Reference Laboratory (Northwest Lipid Metabolism and Diabetes Research Laboratories, University of Washington) to align with the reference monoclonal Lp(a) assay method that is independent of differences in apo(a) isoform size.^29^ Plasma Lp(a) levels were also measured using the polyclonal assay in resurveys (487 participants with repeat assays at 3 years and in 1296 at 8 years after baseline, respectively), allowing assessment of within-person variability in Lp(a) levels by duration of follow-up. Plasma levels of Lp(a) were also measured by a monoclonal assay (reference method), as were plasma apo(a) isoforms using immunoblotting, at the Northwest Lipid Metabolism and Diabetes Research Laboratory in first resurvey samples in 2016 participants.^29^ Comparison of Lp(a) levels obtained by both assay types enabled an assessment of the validity of the polyclonal Lp(a) assay to measure Lp(a) in East Asian populations. Plasma levels of LDL cholesterol (LDL-C) were measured by an immunoturbidimetric assay at the Wolfson Laboratory.

### Genotyping

Genotyping in the CKB study used custom-designed arrays on an Affymetrix Axiom platform,^30^ with content selection similar to that used in UK Biobank (UKB),^31^ but adapted to optimize performance for individuals in East Asian populations. The arrays were designed to maximize genome-wide coverage of common and low-frequency variants in Chinese ancestry populations, detect important variants including rare loss-of-function and other protein-coding variants in Chinese adults, and maintain consistent performance of genotyping across batches of arrays manufactured over an extended time period.^30^ Using 532 415 variants that passed quality control for both array versions, genotyped data were imputed into the 1000 Genomes Phase 3 reference panel, yielding 19 million SNPs with info scores >0.4.

### Follow-up and Disease Phenotyping in CKB

In CKB, both fatal and nonfatal first incident cases of CVD types were ascertained by linkage using a unique identity number to death and disease registries and health insurance records for all hospital admissions. All nonfatal hospitalized cases of first stroke and CHD underwent clinical adjudication by retrieval and review of original medical records and brain imaging records by clinical specialists in China.^32^ In brief, local clinical experts conducted clinical adjudication and defined stroke types (using International Classification of Diseases [ICD]–10 codes of I63 for IS and I61 for intracerebral hemorrhage [ICH] stroke types) in CKB.^32^ Clinical adjudication of 30 000 reported stroke cases using World Health Organization criteria for overall stroke and ICD-11 criteria for IS (including silent lacunar infarcts on brain imaging as IS cases)^28,32^ indicated a reporting accuracy >90% for both stroke types. Likewise, a parallel adjudication study of 37 000 IHD cases indicated a diagnostic accuracy of >98% for reported MI cases.^28^

### Replication of GWAS of Lp(a) in East Asian Individuals in UKB

UKB recruited 502 504 individuals from 22 assessment centers in the United Kingdom between 2006 and 2010. All participants completed a questionnaire, had physical measurements recorded, and provided a blood sample for long-term storage.^31^ Plasma levels of Lp(a) were successfully measured in 460 506 participants (using an identical polyclonal assay as in CKB) and genome-wide genotyping was conducted using an Affymetrix Axiom array. Ethics committee approval to conduct the UKB study was obtained from the North West Multicentre Research Ethics Committee and all participants provided written consent to participate in the study. UKB participants with Chinese or European ancestry were used as replication populations for CKB.

### Combination of CKB With Consortia Data for Transethnic MR Analysis

**Figure 1.**
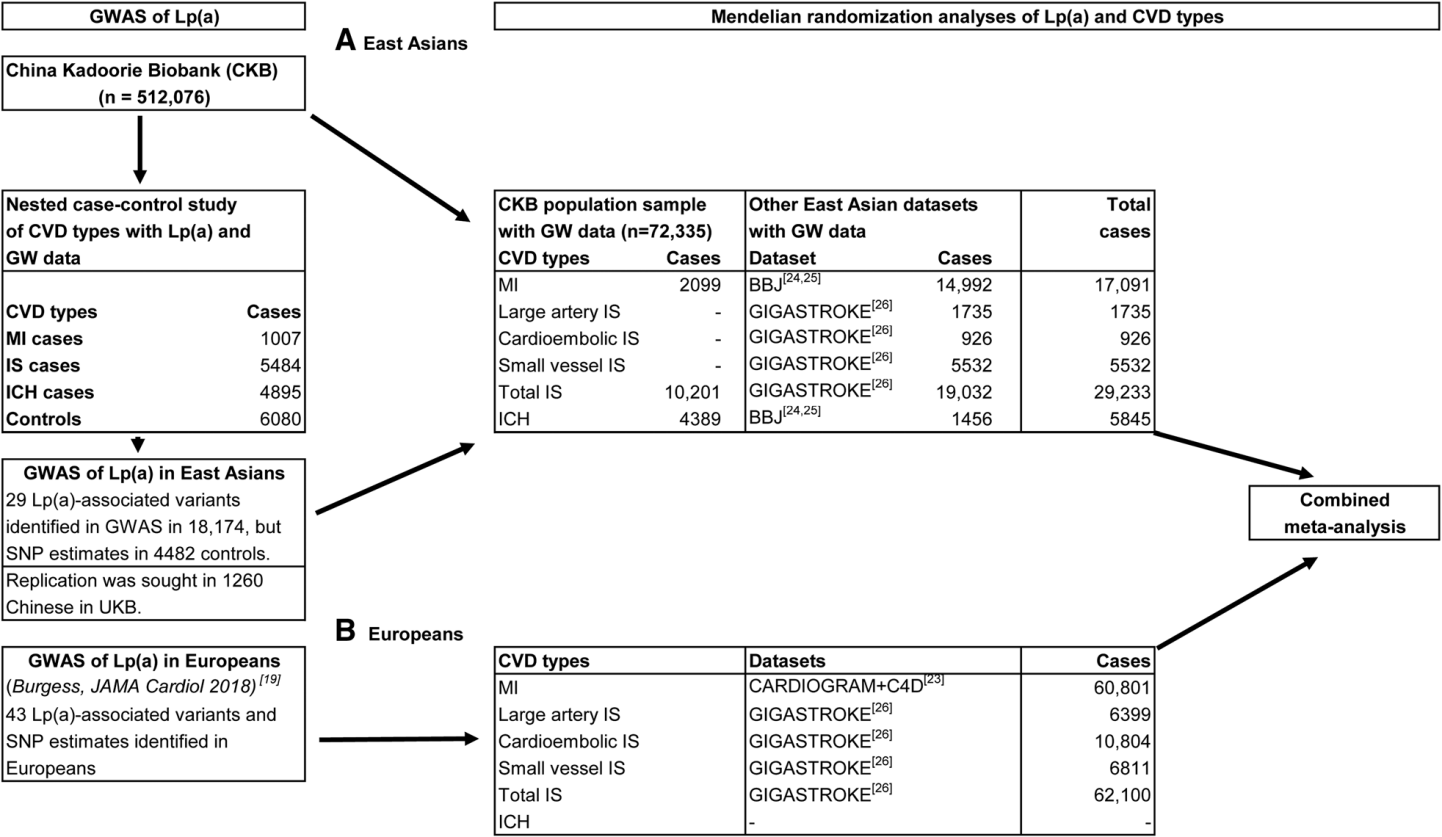
**Study design and number of participants in the genome-wide association study of Lp(a) and in Mendelian randomization studies of Lp(a) and myocardial infarction, ischemic stroke, ischemic stroke subtypes, and intracerebral hemorrhage in East Asian and European individuals. A**, East Asian individuals. **B**, European individuals. BBJ indicates Biobank Japan; CKB, China Kadoorie Biobank; CVD, cardiovascular disease; GW, genome-wide genotyping; GWAS, genome-wide association study; ICH, intracerebral hemorrhage; IS, ischemic stroke; Lp(a), lipoprotein(a); MI, myocardial infarction; MR, Mendelian randomization; SNP, single nucleotide polymorphism; and UKB, UK Biobank.

A nested case–control study of patients with MI, IS, or ICH and shared controls with plasma Lp(a) measurements and genotyping provided data for observational analyses and a GWAS of Lp(a) in CKB (Figure 1). Replication of the GWAS findings in CKB was assessed separately in participants with Chinese or European ancestry in UKB. The variants identified in the GWAS in CKB yielded genetic instruments for MR analyses of Lp(a) in East Asian individuals. MR analyses of genetically predicted Lp(a) with CVD outcomes in CKB were conducted using a population representative sample of 52 552 unrelated participants with genotyping plus additional cases from the nested case–control study (Figure 1 and Table S1).

In addition, 2-sample MR analysis of ancestry-specific Lp(a)-associated variants (using the Burgess et al^19^ SNPs for European individuals) with CHD and stroke types was conducted using publicly available GWAS consortia data from European (CARDIoGRAMplusC4D Consortium and GIGASTROKE) and East Asian populations (BioBank Japan; Figure 1).^24–27^ In GIGASTROKE, IS subtypes were classified using the Causative Classification System into pathogenic subtypes (large-artery atherosclerotic stroke, cardioembolic stroke, and small-vessel IS types),^33^ but there were insufficient numbers of IS subtypes with GWAS data in CKB to include in MR analyses of IS subtypes.

### Statistical Methods

#### Observational Analyses

Lp(a) levels were classified by fifths of the levels in controls, with the upper fifth further subdivided into 2 tenths. Odds ratios (ORs) for vascular disease types by these Lp(a) groups and per 100 nmol/L lower Lp(a) level to simulate the effects of novel Lp(a)-lowering treatments were estimated in a nested case–control study using logistic regression, adjusting for 10 CKB study regions, sex, age, and age^2^ at baseline. Spearman correlation coefficients were used to assess within-person variation in Lp(a) levels between repeat measurements obtained at baseline and sequential resurveys.

#### GWAS for Lp(a) in CKB

Participants without valid Lp(a) measurements were excluded, but there were no other exclusions for missing covariates. For GWAS analyses of Lp(a) among 18 174 genotyped participants with Lp(a) measurements, Lp(a) was transformed by rank-inverse normal transformation of the residual log Lp(a) levels after regression on age, age^2^, sex, and study region. GWAS of transformed Lp(a) was conducted with linear mixed models using BOLT-LMM (version 2.3.2), which accounts for relatedness between individuals.^34^ Genomic regions surrounding SNPs with *P*<5×10^−^^8^ were delineated by identifying SNPs with linkage disequilibrium r^2^>0.05 within ±5 000 000 base pairs using PLINK v1.07. Independent association signals within these regions were then identified using conditional and joint analysis with genome-wide complex trait analysis conditional and joint analysis software.^35^ Among the 29 SNPs with a minor allele frequency ≥0.5% that were conditionally independently associated with Lp(a) levels at genome-wide significance (*P* <5×10^−^^8^), 28 SNPs were at the *LPA* locus on chromosome 6 and 1 SNP was at the *APOE* locus on chromosome 19.

#### Associations of Genetic Variants With Measured Lp(a)

To estimate the effects of these SNPs on untransformed Lp(a) levels, the per allele effects of each SNP on Lp(a) were estimated among 4482 unrelated controls with Lp(a) levels at baseline and genetic data separately in each region using linear regression models with adjustment for sex, age, age^2^, and up to 9 region-specific genetic principal components, and were then combined across regions using inverse-variance weighted meta-analysis. In UKB participants,^31^ the effects of the 28 of these 29 Lp(a)-related SNPs that were available were estimated separately in 1260 unrelated Chinese and 329 968 unrelated White British participants using linear regression models and adjusted for sex, age, age^2^, and 40 principal components. Similar approaches were used to estimate associations with Lp(a) in CKB using SNPs previously reported for European^19^ and East Asian^22^ populations (Methods in the Supplemental Material). The variance of Lp(a) explained by sets of SNPs was estimated using the partial R^2^ method. To assess the associations of these variants with baseline characteristics in CKB, a genetic score based on the 29 SNPs discovered in CKB was constructed. The genetically predicted Lp(a) values for each participant were the fitted values from a regression of measured Lp(a) on the Lp(a)-associated genetic score (Methods in the Supplemental Material).

#### MR analyses

Inverse-variance weighted MR was used to estimate the causal relevance of 100 nmol/L lower genetically predicted Lp(a) in CKB taking account of linkage disequilibrium between SNPs, using the MendelianRandomization R package.^36–38^ The hazard ratios (HRs) and 95% CIs for associations of genetically predicted Lp(a) with CVD outcomes in CKB were estimated using the Prentice case–cohort extension^39^ of the Cox proportional hazards model (which allows for analyses of a combination of cases and a random selection of the study population for a case–cohort design), stratified by sex and 5-year age-at-risk groups (age at risk, 40–79 years) and adjusted for 9 region-specific principal components of ancestry. Only a modest proportion of participants used in the case–cohort MR analyses of genetically predicted Lp(a) with CVD outcomes (≈5% for major vascular events) overlapped with those in the SNP-Lp(a) effect analyses (Table S1). In sensitivity analyses, CVD outcome MR analyses were repeated excluding the participants used to estimate SNP associations, separately, with measured Lp(a) (Methods in the Supplemental Material and Table S1) and restricting the Lp(a) instrument to the 28 SNPs at the *LPA* locus.

Further ancestry-specific MR analyses of CVD outcomes were conducted using CKB data and publicly available GWAS consortia summary data in Figure 1.^24–27^ In the East Asian ancestry analyses, the 29 Lp(a)-associated variants identified in this study and their effect sizes on Lp(a) in CKB were used. In the European ancestry analyses, 43 Lp(a)-associated variants and their effect sizes identified in a previously reported GWAS meta-analysis of 48 333 European individuals were used.^19^ For comparative analyses in East Asian and European individuals, the HRs for MI or stroke types in CKB and corresponding ORs for CHD or stroke from GWAS consortia were labeled as rate ratios (RRs) for 100 nmol/L lower genetically predicted Lp(a) (Methods in the Supplemental Material). The genetic analyses were reported in accordance with guidelines for reporting of observational studies using Mendelian randomization (Strengthening the Reporting of Observational Studies in Epidemiology using Mendelian Randomization).^40^ All statistical analyses were performed in R (version 4.2.2), including the MendelianRandomization R package for standard MR analyses and MR analyses by robust methods (Methods in the Supplemental Material).

## RESULTS

### Distribution of Lp(a) and apo(a) Isoforms in Chinese Adults

**Figure 2.**
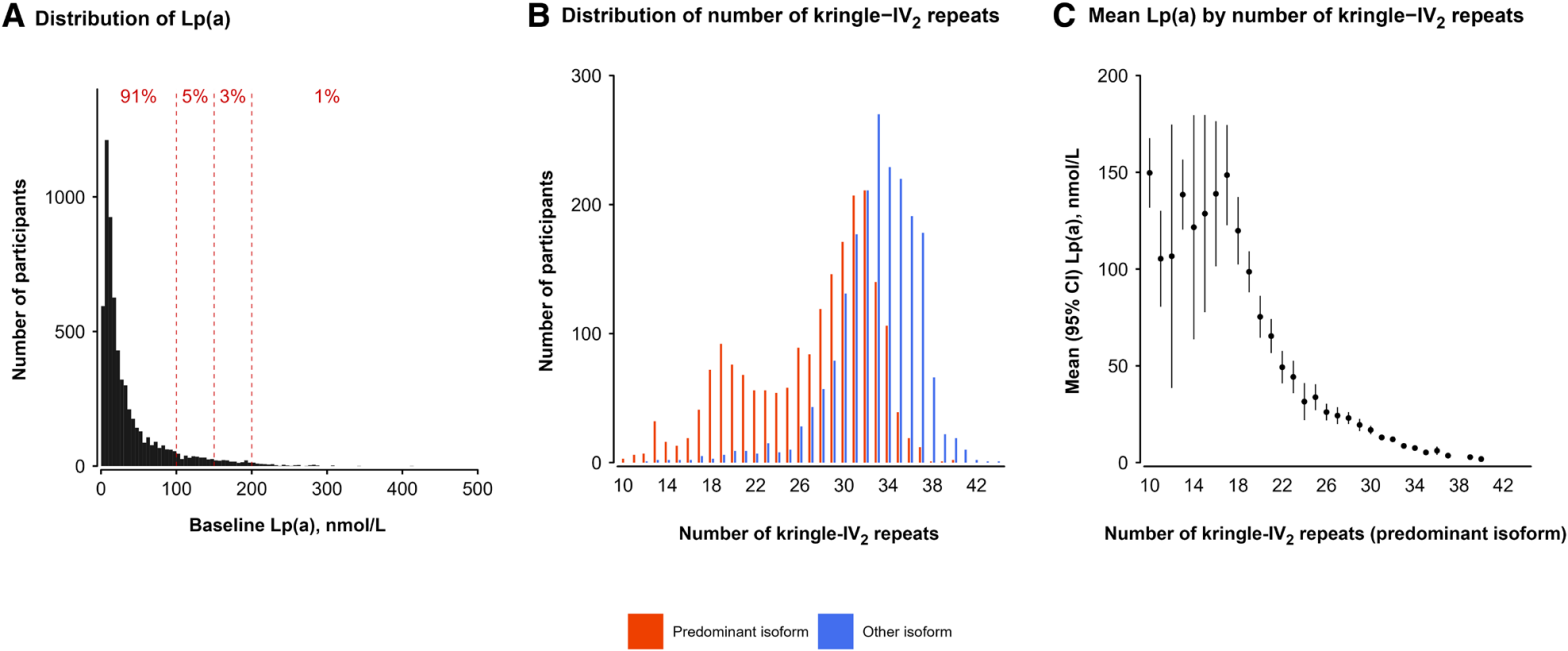
**Distribution of Lp(a) and apo(a) isoforms and associations of Lp(a) levels with apolipoprotein(a) isoforms in China Kadoorie Biobank. A**, The distribution of Lp(a) [lipoprotein(a)] in 6339 control participants. **B**, apo(a) [apolipoprotein(a)] isoforms (kringle IV type 2 repeats) in 2016 participants. **C**, Associations of Lp(a) levels with apo(a) isoforms in 1324 control participants with isoform data.

In CKB, the distribution of plasma levels of Lp(a) in controls was highly right-skewed, and only 8% of controls had Lp(a) levels ≥100 nmol/L and 4% Lp(a) levels ≥150 nmol/L (Figure 2A). The median levels of Lp(a) in Chinese individuals were comparable with those in European individuals in UKB,^3^ but the proportions with Lp(a) levels ≥100 nmol/L were 2-fold greater in European individuals than in Chinese individuals.^3^ In a subset of participants, the Spearman correlation coefficients (95% CI) between Lp(a) levels at baseline and repeat measurements 3 and 8 years later assessed using an identical polyclonal Lp(a) assay were 0.93 (0.92–0.95) and 0.91 (0.89–0.92), respectively (Figure S1). The agreement in plasma Lp(a) levels measured using the polyclonal assay at baseline and a monoclonal assay at first resurvey yielded a Spearman correlation coefficient (95% CI) of 0.92 (0.91–0.92) (Figure S1). There were minor deviations in the accuracy of the polyclonal Lp(a) assay at Lp(a) levels ≥150 nmol/L. apo(a) isoforms in Chinese adults had a bimodal distribution, with ≈25% having a small predominant isoform with ≤22 KIV2 repeats (Figure 2B). Plasma levels of Lp(a) in CKB were inversely associated with number of KIV2 repeats, consistent with findings in European individuals (Figure 2C).^4,5^

### Observational Associations of Lp(a) With CHD and Stroke

**Figure 3.**
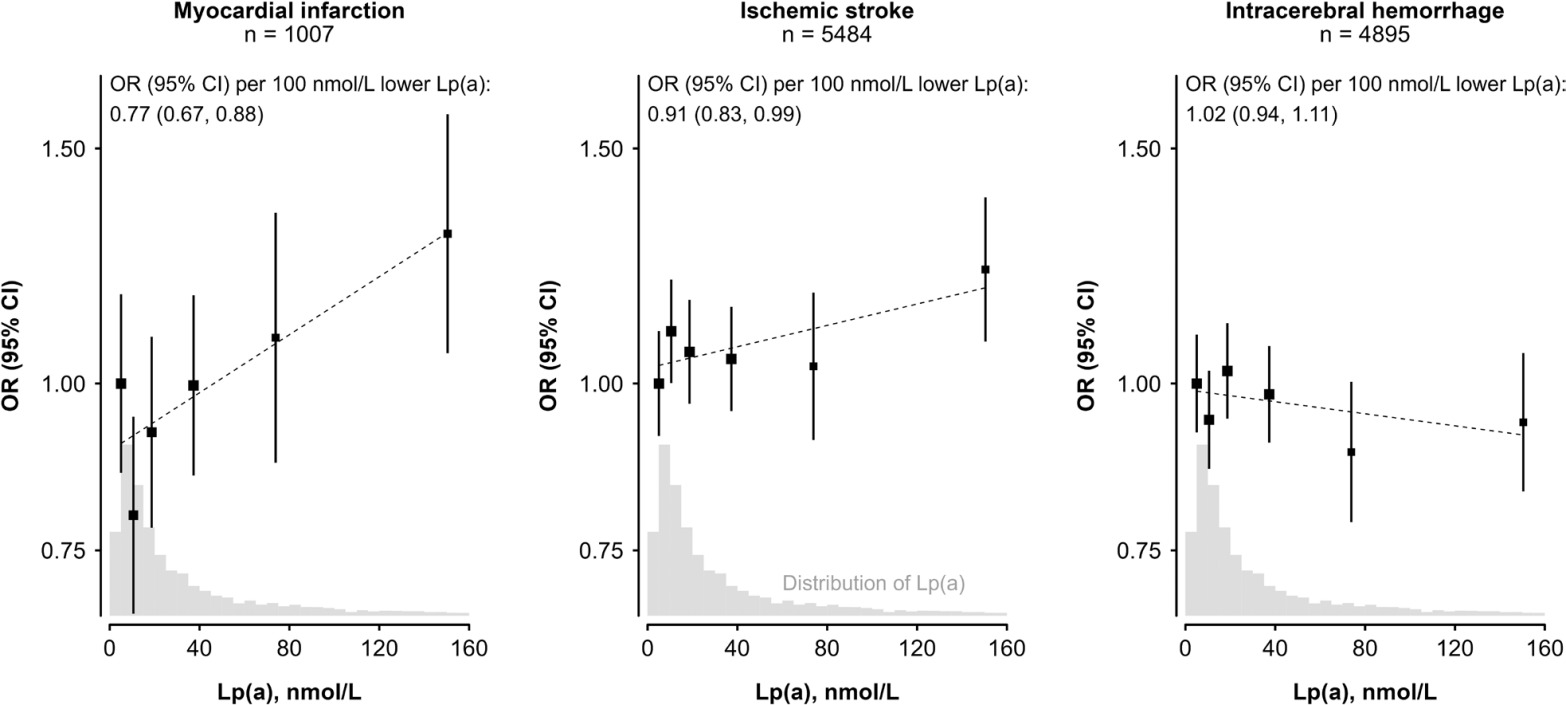
**Observational associations of plasma levels of Lp(a) with risk of myocardial infarction, ischemic stroke, and intracerebral hemorrhage in China Kadoorie Biobank.** The values shown are odds ratios (ORs) and 95% CIs for myocardial infarction (left), ischemic stroke (center), and intracerebral hemorrhage (right) for fifths of Lp(a) [lipoprotein(a)] relative to the bottom fifth (reference group). Values in the top fifth were subdivided into 2 tenths. Analyses used 6339 control participants plus incident cases (numbers in subtitles). *P* values for tests of nonlinearity among the 6 groups for all disease outcomes were nonsignificant.

In CKB, the mean age of patients with MI (n=1007) or IS (n=5484) was lower than that of controls (n=6080) or patients with ICH (n=4895). The mean±SD ages of patients (MI, 53±7 years; IS, 54±9 years; ICH, 59±10 years) were comparable with that of controls (59±10 years), as were the proportions of women in each group (62%, 47%, 52%, 52%, respectively). The MI, IS, and ICH groups included a higher proportion of current cigarette smokers and patients with diabetes, and had higher mean levels of systolic blood pressure than in the controls. With the groups with the lowest levels of Lp(a) as a reference group, there were log-linear positive associations of higher levels of Lp(a) with higher risks of MI and IS (Figure 3). However, the strengths of the associations per 100 nmol/L lower Lp(a) with MI were 3-fold greater than with IS (OR, 0.77 [95% CI, 0.67–0.88] versus 0.91 [0.83–0.99], respectively). By contrast, Lp(a) levels were unrelated to risk of ICH (1.02 [0.94–1.11]). The strength of association of Lp(a) with MI was unaltered by additional adjustment for established CVD risk factors (Figure S2).

### Genetic Determinants of Lp(a) in Chinese Adults

**Figure 4.**
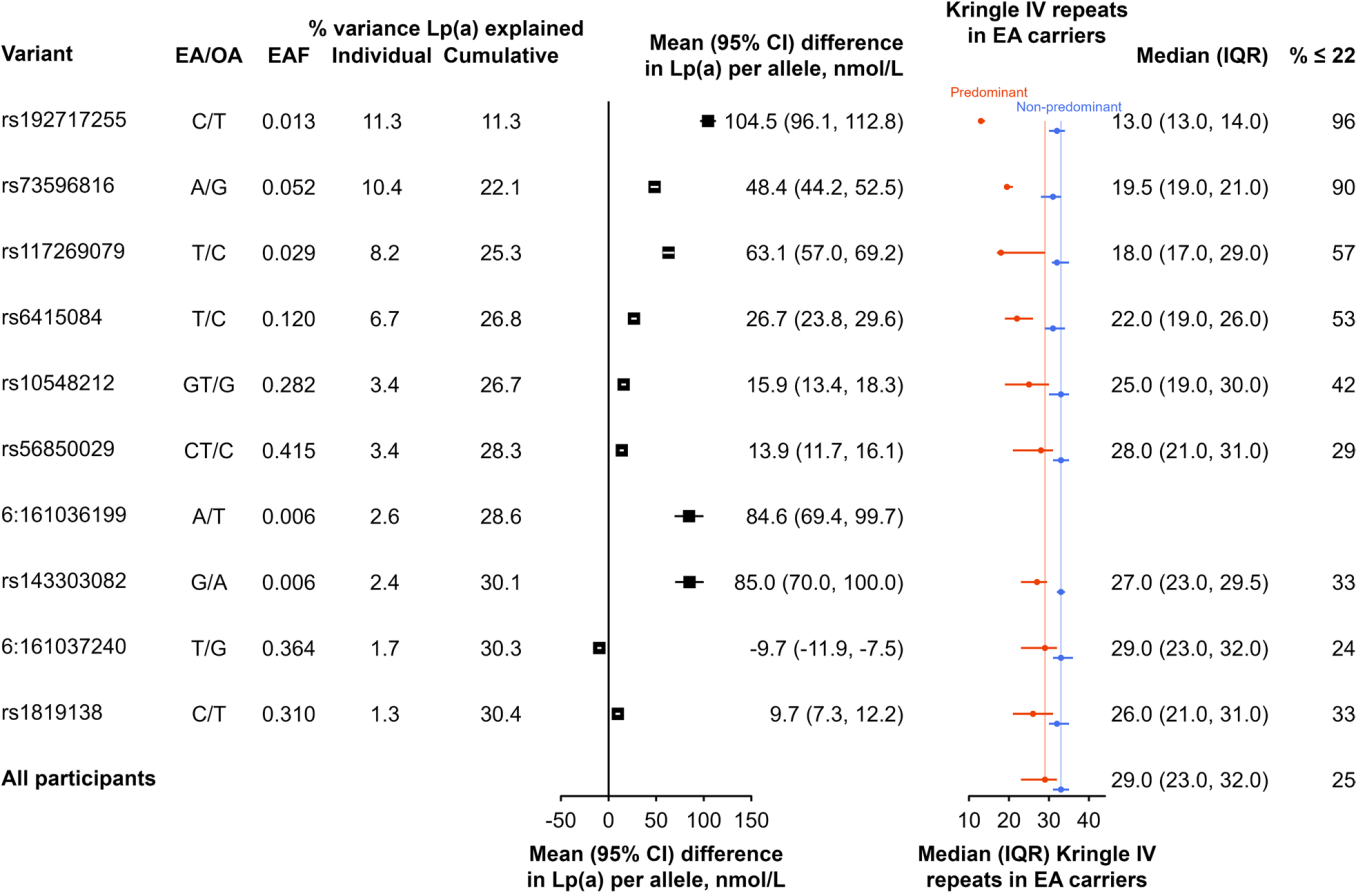
**Associations of LPA genetic variants with plasma levels of Lp(a) and apo(a) isoform size in Chinese adults in China Kadoorie Biobank.** The values shown are regression coefficients (95% CI) for effects of individual SNPs on Lp(a) [lipoprotein(a)] estimated in 4482 control participants with genetic data (excluding related participants and immigrants; left) and median (interquartile range [IQR]) kringle IV repeats in 2016 participants with isoform data (right). Rows were ordered by decreasing percentage of variance in Lp(a) levels explained by different the variants. Median kringle IV repeats refer to the predominant apo(a) [apolipoprotein(a)] isoforms. EA indicates effect allele; EAF, effect allele frequency; and OA, other allele.

SNPs used as Lp(a) genetic instruments for MR analyses were derived as conditionally independent association signals from GWAS results in CKB (Table S2) with effects on Lp(a) estimated in controls (Table S3). This process identified 29 SNPs (28 at the *LPA* locus and 1 at the *APOE* locus; Table S2) and details for 10 SNPs that explained most variance in Lp(a) levels are shown in Figure 4 (and for all 29 SNPs in Figure S3). All 29 SNPs associated with Lp(a) together explained 33% of the variance in Lp(a) in CKB (Figure S3), with 2 genetic variants (rs73596816 and rs192717255) each accounting for ≈11% of variance. The 10 variants accounting for most of the variance of Lp(a) levels in CKB showed directionally consistent (*P*<0.05) associations with Lp(a) in the 1260 Chinese adults in UKB,^31^ providing replication in an independent population (Figure S3 and Table S4). The chromosomal locations for the identified variants at the *LPA* locus in CKB are shown in a locus zoom plot in Figure S4.

The SNPs most strongly associated with Lp(a) in CKB had the smallest median apo(a) isoforms; the top 4 *LPA* variants ranked by variance explained in Lp(a) each had median KIV2 ≤22 (Figure 2). The distributions of the KIV2 repeats associated with each of the 29 SNPs are shown in Figure S5. Table S5 shows mean levels of CVD risk factors separately by plasma levels of Lp(a) and genetically predicted Lp(a), indicating modest associations of genetically predicted Lp(a) with LDL-C and apolipoprotein B, but not with the other CHD risk factors.

We further assessed the associations of SNPs previously identified at the *LPA* locus^4,19^ or outside the *LPA* locus^20^ in European individuals and East Asian individuals with Lp(a) in CKB (Table S6). We also assessed the associations of the SNPs identified in CKB with Lp(a) in White British individuals in UKB^31^ (Table S7), and assessed linkage disequilibrium (LD) in CKB between the CKB-identified SNPs and previously identified SNPs. These between-ancestry comparisons showed that Lp(a)-associated variants in either European individuals or East Asian individuals had limited associations with Lp(a) in the other ancestry (Figures S6 and S7 and Tables S4, S6, and S7). The chief European *LPA* SNPs had negligible effects on Lp(a) levels in CKB, with rs10455872 being very rare in Chinese individuals and rs3798220 having only minimal effects on Lp(a), and these SNPs were not in LD with any other SNPs identified in CKB (Tables S6 and S8). One SNP, rs73596816, explaining 10.4% of the variance in Lp(a) in CKB, was identified by Burgess et al^19^ and Liu et al^22^ and, in addition, the other 3 SNPs reported by Liu et al^22^ in a Chinese population were replicated in CKB (and 2 of these had LDs of r^2^=0.83 and 0.58 with other SNPs identified in CKB; Table S9). The 4 SNPs identified by Liu et al^22^ explained 13.2% of the variance of Lp(a) in CKB. Two other SNPs discovered by Burgess et al^19^ were also in LD with SNPs identified in CKB, with r^2^ of 0.97 and 0.74 (Table S10). The 2 additional SNPs outside the *LPA* locus associated with Lp(a) in Hoekstra et al^20^ and with minor allele frequency ≥0.05% in CKB were not statistically significantly associated with Lp(a) in CKB (Table S6 and Figure S6). In contrast with the 43 European SNPs (discovered by Burgess et al^19^) that explained 62% of variance in Lp(a) in the current analysis of Lp(a) in UKB, 28 of the 29 Chinese SNPs that were also available in UKB explained only 10% of the variance in Lp(a) in UKB, and adding the Chinese to the European SNPs did not alter the variance in Lp(a) explained by the European SNPs in European individuals. Adding European Lp(a)-associated SNPs to Chinese SNPs did not alter the variance in Lp(a) levels explained by Chinese SNPs in Chinese individuals.

### MR Analysis of Genetically Predicted Lp(a) With CHD and Stroke in CKB

In CKB, the HRs (95% CI) per 100 nmol/L lower genetically predicted Lp(a) were 0.82 (0.70–0.96) for acute MI and 0.81 (0.69–0.95) for CHD death (Figure S8), consistent with the findings of the observational analyses. By contrast, there were no significant associations of genetically predicted Lp(a) with IS (0.95 [0.88–1.03]), vascular death (0.97 [0.91–1.02]), or any major vascular events (0.97 [0.91–1.02]) in CKB. In sensitivity analyses, after excluding overlapping participants used to estimate SNP effects, the results were largely unaltered (Table S11). After excluding the rs1065853 SNP at the *APOE* locus, the results for a genetic score involving 28 SNPs at the *LPA* locus alone did not differ from those for all 29 SNPs associated with Lp(a) levels used in the main analyses for this report (Table S12).

### MR Analyses of Lp(a) With CHD and Stroke Types in Global GWAS Consortia

**Figure 5.**
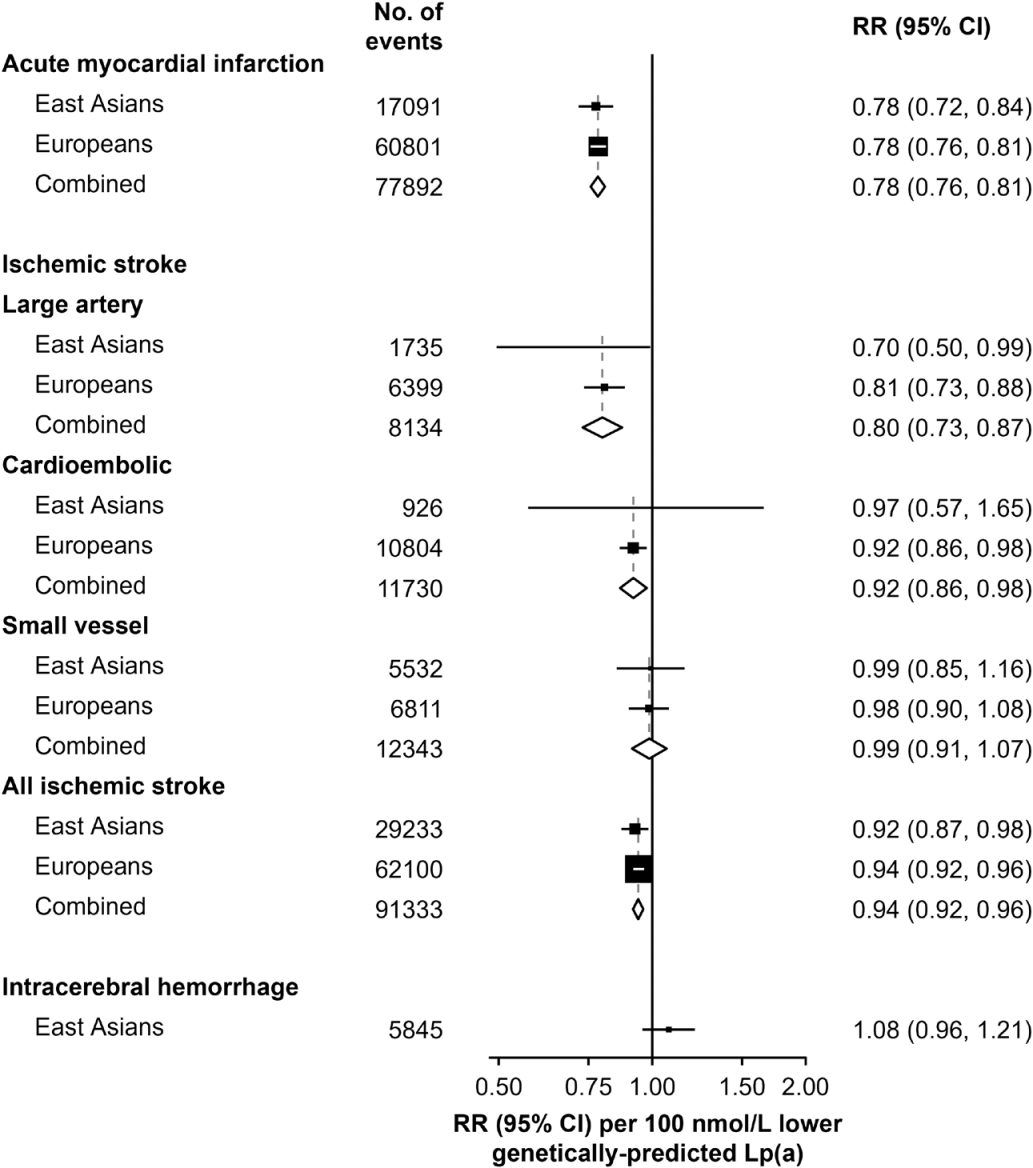
**Comparison of 100 nmol/L lower genetically predicted Lp(a) levels on risk of myocardial infarction, ischemic stroke, and intracerebral hemorrhage in East Asian and European populations.** The values shown are rate ratios (RRs) with 95% CIs for 100 nmol/L lower Lp(a) [lipoprotein(a)] on myocardial infarction, ischemic stroke, ischemic stroke subtypes, and intracerebral hemorrhage in East Asian and European individuals and all participants. Additional details of the studies and numbers of cases and controls for each outcome are provided in Table S4.

In ancestry-specific 2-sample MR analyses of MI (n=77 892), IS (n=91 333), and ICH (n=5845) cases in East Asian individuals and European individuals (Table S13), there were concordant RRs per 100 nmol/L lower Lp(a) in East Asian individuals and European individuals for risk of MI (0.78 [95% CI, 0.72–0.84] versus 0.78 [0.76–0.81], respectively) and large-artery IS (0.78 [0.76–0.81] versus 0.80 [0.73–0.87], respectively) (Figure 5). The RRs for all other outcomes were concordant in East Asian individuals (mainly BioBank Japan) and European individuals, but the overall effects on cardioembolic IS were much weaker (0.92 [0.86–0.98]), and were not statistically significant for small-vessel IS (0.99 [0.91–1.07]) or ICH (1.08 [0.96–1.21] in East Asian individuals only). Results of alternative MR approaches involving different assumptions (Methods in Supplemental Material) were unaltered, providing support for the validity of the causal relevance of genetically predicted Lp(a) for MI and stroke types (Table S14).

## DISCUSSION

In CKB, GWAS analyses of plasma levels of Lp(a) identified 29 Lp(a)-associated SNPs that accounted for 33% of the variance in Lp(a) levels in this Chinese population and these showed only modest overlap in the genetic variants for Lp(a) and their effects on Lp(a) levels between Chinese and European individuals. The GWAS findings for Lp(a) in CKB were replicated in participants with Chinese ancestry in UKB. Using ancestry-specific genetic instruments, combined MR analyses involving CKB with other East Asian and European consortia showed that the effects of 100 nmol/L lower genetically predicted Lp(a) on risk of MI were comparable between East Asian and European individuals. Moreover, the effects on MI were 3-fold greater than on total IS, but similar for large-artery IS in both ancestries. Observational analyses in CKB were also consistent in showing stronger associations of Lp(a) levels with MI than with total IS.

Despite differences in the identity of the Lp(a)-associated variants between European and East Asian individuals, there was a modest agreement in effect sizes of individual variants between ancestries, but the results highlight the need to use ancestry-specific instruments for MR analyses of Lp(a) with risk of MI and stroke subtypes in East Asian and European individuals. Nevertheless, the current report found that the effects of equivalent absolute differences in genetically predicted Lp(a) on risk of MI and stroke types and on IS subtypes were concordant in East Asian and European individuals. Moreover, the genetic variants identified in CKB demonstrated comparable strength of associations with MI in Japanese (BioBank Japan) and Chinese (CKB) participants, providing support for their use as instrumental variables for MR analyses of Lp(a) in East Asian individuals. As in European individuals,^4,5^ the associations of the 29 Lp(a)-associated SNPs with apo(a) isoforms identified in CKB showed that SNPs with the greatest effects on Lp(a) were associated with the smallest apo(a) isoforms. The bimodal distributions of apo(a) isoforms observed in CKB were consistent with those previously reported in Chinese participants in the HPS-2 THRIVE trial population.^41^ The current report also demonstrated comparable accuracy of the currently available polyclonal Lp(a) assay with a monoclonal antibody assay that is independent of apo(a) isoform size for detection of individuals with elevated levels of Lp(a) in East Asian populations.

The chief strengths of this study include the findings of substantial differences in effects of genetically predicted Lp(a) levels on risk of MI versus IS (22% versus 8% lower risk per 100 nmol/L lower genetically predicted Lp[a], respectively) and among the different IS subtypes (20% versus 8% versus 0% for large-artery IS, cardioembolic IS, and small-vessel IS per 100 nmol/L lower genetically predicted Lp[a], respectively) that were concordant between ancestries. Whereas previous observational studies had reported differences in mean levels of Lp(a) among IS subtypes,^42,43^ the large number of IS subtypes and stronger instrumental variables for MR analyses provided reliable evidence for their causal relevance. Moreover, the MR results for effects of genetically predicted Lp(a) on MI, IS, and IS subtypes were qualitatively consistent with those previously reported for LDL-C,^44,45^ with greater effects on MI than on IS, but similar effects on large-artery IS, but not on cardioembolic or small-vessel IS subtypes.^43–45^

This study had several limitations, including restriction of data on Lp(a) levels to subsets of CKB participants and limited numbers of ICH cases. Whereas the observational and genetic associations of Lp(a) with ICH were not statistically significant, the 95% CIs for the RR per 100 nmol/L lower genetically predicted Lp(a) for ICH in CKB were compatible with a higher risk of ICH, consistent with the findings for LDL-C and ICH.^46^

The concordant RRs for 100 nmol/L lower genetically-predicted Lp(a) for MI in East Asian individuals and European individuals would suggest that both populations should expect comparable benefits for prevention of MI for equivalent reductions in Lp(a) by use of potent novel Lp(a)-lowering medications. However, because the strength of association of elevated level of Lp(a) with IS was only one-third of that for MI and involved mainly large-artery IS subtypes, it is likely that the elevated levels of Lp(a) are more strongly related to atherosclerosis than to thrombosis. The biologic function of Lp(a) in healthy adults is not fully understood, but the mechanisms by which elevated levels cause vascular disease may reflect their carriage of oxidized phospholipids^47,48^ or inhibition of plasminogen and promotion of thrombosis.^49^ The findings of this study highlight the role of Lp(a) for large-artery atherosclerosis, and suggest that Lp(a) may be relevant to peripheral arterial disease or heart failure that were not examined in this study.

MR studies assessing effects of lowering Lp(a) levels on CHD and stroke subtypes can inform the design and conduct of randomized trials^8,9,12,13,50–52^ where the primary outcomes are restricted to major coronary events for some trials, but others involve a composite of major coronary events, stroke, and other vascular events. MR studies assess effects of lifelong differences in Lp(a) in general populations, but trials assess effects of short-term reductions in Lp(a) in high-risk participants.^53^ Therefore, results of MR studies of Lp(a) may not directly anticipate the effects of treatment in Lp(a)-lowering trials, especially for composite outcomes (including stroke and CHD), where the proportions of IS subtypes differ between East Asian and European individuals. Whereas MR analyses indicated weaker effects of LDL-C on IS, randomized trials of statin therapy conducted in European ancestry populations reported comparable proportional reductions on CHD and IS events (but data on IS subtyping were not reported in the large meta-analyses of such trials).^54,55^ By contrast with previous reports,^56,57^ the current study suggests that for CHD the effects of genetically predicted 100 nmol/L lower Lp(a) levels were comparable with those for 1 mmol/L lower LDL-C level achieved by statins in the Cholesterol Treatment Trialists’ Collaboration (22% [95% CI, 19–24] versus 24% [21–27]),^58^ but those for stroke were lower than the reductions achieved by statins (6% [4–8] versus 15% [11–20], respectively).^58^

The Cholesterol Treatment Trialists’ Collaboration demonstrated that the benefits of statins were relevant to all participants irrespective of pretreatment LDL-C levels,^58^ but as the distribution of Lp(a) is highly skewed in both ancestry populations, the relevance of novel Lp(a)-lowering treatments is likely to be limited to those with plasma levels of Lp(a) >100 nmol/L, which are more frequent in European than in East Asian individuals.^3^ The Beijing Heart Society Expert Scientific Statement^59^ on management of Lp(a) levels for cardiovascular disease prevention in Chinese adults recently recommended measuring Lp(a) levels at least once in patients with early onset atherosclerotic CVD. The findings of the current study provide additional support for prioritizing Lp(a) measurements in East Asian as well as European patients with early onset MI or large-artery IS subtypes.

Three ongoing trials of novel ASO or siRNA therapies that lower Lp(a) levels by >90% (Table S15) involving 28 120 European and East Asian participants^50–52^ are assessing the relevance of lowering Lp(a) levels on risk of CHD or stroke (or a composite of these outcomes). The discrepant results of MR studies of Lp(a) for IS subtypes highlight the importance of subtyping of IS outcomes in these Lp(a)-lowering trials.^50–52^ Assuming that the efficacy and safety of such novel potent Lp(a)-lowering treatments on MI (and some stroke outcomes) are confirmed, this study provides support for extending a precision medicine approach to prioritizing such treatments to high-risk East Asian as well as European patients with high plasma levels of Lp(a).

## ARTICLE INFORMATION

### Acknowledgments

The authors thank the participants and CKB project staff for their participation; staff of the China Center for Disease Control and Prevention and its regional offices for access to death and disease registries; and Professors Jemma Hopewell and David Preiss for discussion. The Chinese National Health Insurance scheme provided electronic linkage to all hospital admissions. The CKB study is jointly coordinated by the University of Oxford and the Chinese Academy of Medical Sciences. This research was conducted using the UK Biobank Resource under application number 50474.

### Sources of Funding

The funding body for the baseline survey was the Kadoorie Charitable Foundation, Hong Kong, China, and the funding sources for the long-term continuation of the study include UK Wellcome Trust (grants 202922/Z/16/Z, 104085/Z/14/Z, and 088158/Z/09/Z), the National Natural Science Foundation of China (grants 82192900, 82192901, 82192904, and 82388102), and the Noncommunicable Chronic Diseases–National Science and Technology Major Project (grant 2023ZD0510100). Core funding was provided to the CTSU, University of Oxford, by the British Heart Foundation, the UK Medical Research Council, and Cancer Research UK. The long-term follow-up was funded in part by the UK Wellcome Trust (grants 212946/Z/18/Z, 202922/Z/16/Z, 104085/Z/14/Z, and 088158/Z/09/Z).

### Disclosures

None.

### Supplemental Material

Methods

Figures S1–S8

Tables S1–S15

Checklist

Appendix

References 60–64
